# A Novel Adeno-Associated Viral Variant for Efficient and Selective Intravitreal Transduction of Rat Müller Cells

**DOI:** 10.1371/journal.pone.0007467

**Published:** 2009-10-14

**Authors:** Ryan R. Klimczak, James T. Koerber, Deniz Dalkara, John G. Flannery, David V. Schaffer

**Affiliations:** 1 Department of Molecular and Cellular Biology, The University of California, Berkeley, California, United States of America; 2 Department of Chemical Engineering and the Department of Bioengineering, The University of California, Berkeley, California, United States of America; 3 The Helen Wills Neuroscience Institute, The University of California, Berkeley, California, United States of America; National Institute on Aging, United States of America

## Abstract

**Background:**

The pathologies of numerous retinal degenerative diseases can be attributed to a multitude of genetic factors, and individualized treatment options for afflicted patients are limited and cost-inefficient. In light of the shared neurodegenerative phenotype among these disorders, a safe and broad-based neuroprotective approach would be desirable to overcome these obstacles. As a result, gene delivery of secretable-neuroprotective factors to Müller cells, a type of retinal glia that contacts all classes of retinal neurons, represents an ideal approach to mediate protection of the entire retina through a simple and innocuous intraocular, or intravitreal, injection of an efficient vehicle such as an adeno-associated viral vector (AAV). Although several naturally occurring AAV variants have been isolated with a variety of tropisms, or cellular specificities, these vectors inefficiently infect Müller cells via intravitreal injection.

**Methodology/Principal Findings:**

We have previously applied directed evolution to create several novel AAV variants capable of efficient infection of both rat and human astrocytes through iterative selection of a panel of highly diverse AAV libraries. Here, in vivo and in vitro characterization of these isolated variants identifies a previously unreported AAV variant ShH10, closely related to AAV serotype 6 (AAV6), capable of efficient, selective Müller cell infection through intravitreal injection. Importantly, this new variant shows significantly improved transduction relative to AAV2 (>60%) and AAV6.

**Conclusions/Significance:**

Our findings demonstrate that AAV is a highly versatile vector capable of powerful shifts in tropism from minor sequence changes. This isolated variant represents a new therapeutic vector to treat retinal degenerative diseases through secretion of neuroprotective factors from Müller cells as well as provides new opportunities to study their biological functions in the retina.

## Introduction

As a therapeutic device, adeno-associated viral (AAV) mediated ocular gene therapy holds tremendous promise for treating and potentially curing a variety of inherited retinal degenerative diseases, such as glaucoma, age-related macular degeneration (AMD), retinitis pigmentosa, and other blinding diseases. The therapeutic potential of AAV-mediated gene delivery to the retina has best been illustrated by the positive results from three phase I clinical trials for Leber's Congenital Amaurosis (LCA), a rare autosomal recessive blinding disease caused by loss of function mutations in the visual cycle enzyme retinol isomerase (RPE65) required to synthesize 11-cis retinal [1]–[3]. Gene-replacement therapy employing an AAV vector (rAAV) bearing a functional copy of the RPE65 gene in patients afflicted with the null mutant RPE65 resulted in significant improvements in visual function with no observable toxicity. These clinical trials clearly lay the foundation for AAV in retinal gene therapy, providing the first example of successful gene therapy with long-term safety and amelioration in visual function. Moreover, these results set the stage for the application of AAV-mediated gene delivery towards other retinal degenerative diseases.

Importantly, genetic heterogeneity is a key feature of retinal degeneration conditions, and to date, over 130 genes with mutations causing one or more forms of inherited orphan retinal degenerative diseases have been cloned, and over 50 more have been identified based on candidate gene studies or linkage mapping (http://www.sph.uth.tmc.edu/retnet). This large genetic heterogeneity, leading to a common phenotype of photoreceptor cell death, suggests that a mutation-independent neuroprotective strategy may be the most practical solution to a clinical treatment. Therefore, there is a strong need for a simple, safe, and broad-based ocular gene delivery strategy that provides sustained production of a neuroprotective or anti-angiogenic therapeutic for these conditions.

Several viral vector systems have been evaluated for ocular gene delivery; however, AAV offers distinct advantages in safety, stability, and efficiency. AAV belongs to the *Parvoviridae* family and Dependovirus genus, whose members require co-infection with a helper virus such as adenovirus to promote replication, and AAV establishes a latent infection in the absence of a helper [4]. Virions are composed of a 25 nm icosahedral capsid encompassing a 4.9 kb single-stranded DNA genome [5] with two open reading frames: *rep* and *cap*. The non-structural *rep* gene encodes four regulatory proteins essential for viral replication, whereas *cap* encodes three structural proteins (VP1–3) that assemble into a 60-mer capsid shell. This viral capsid mediates the ability of AAV vectors to overcome many of the biological barriers of viral transduction–including cell surface receptor binding, endocytosis, intracellular trafficking, and unpackaging in the nucleus [6]–[8]. In addition, the capsid diversity found among natural AAV variants, or serotypes, isolated from human and nonhuman primate tissues accounts for its diverse tropisms, as AAV has been shown in animal models to deliver genes to cells in numerous tissues including the retina, brain, muscle, and lung [9]–[12]. As some AAV serotypes bind to glycans for receptor-mediated cellular entry, this variety is in part based on the varying glycan dependences found among AAV serotypes. AAV2 shows strong dependence on heparan sulfate proteoglycans (HSPG) for transduction, whereas AAV1, 5, and 6 are dependent on N-linked sialic acids [4], [5], [13].

Subretinal administration of many of these AAV variants has led to efficient gene expression in a number of retinal neurons and epithelia, including photoreceptors and RPE [14], [15], and it is thus the injection route employed by current clinical therapies to achieve sufficient transduction for rescue [2]. However, this surgical approach requires creating a retinotomy (a hole through the neurosensory retina) and mechanically detaching the photoreceptor layer from its underlying supportive epithelium (RPE) through injection of an AAV fluid suspension, generating a “bleb”. The resulting retinal detachment has had documented damaging effects, triggering cellular stress response pathways, reactive gliosis, retinal disorganization, photoreceptor degeneration, and functional losses in vision [16], [17]. In retinas compromised by degeneration, the effects of this detachment would only be further magnified [18]. Additionally, as degeneration occurs throughout the retina in most retinal diseases, use of a focal delivery and treatment strategy via subretinal injection into a specific region is not optimal because only cells within the “bleb” are transduced [19]–[21]. An intravitreal injection technique, whereby virus is administered directly into the vitreous of the eye, presents a more innocuous and simple approach for gene delivery to the retina, and it allows for a broader area of retinal transduction since the vitreous contacts the entire underlying retinal surface. Intravitreal injection is already routinely used clinically to administer anti-vascular endothelial growth factor (anti-VEGF) antibody for the treatment of AMD [22].

**Figure 1:**
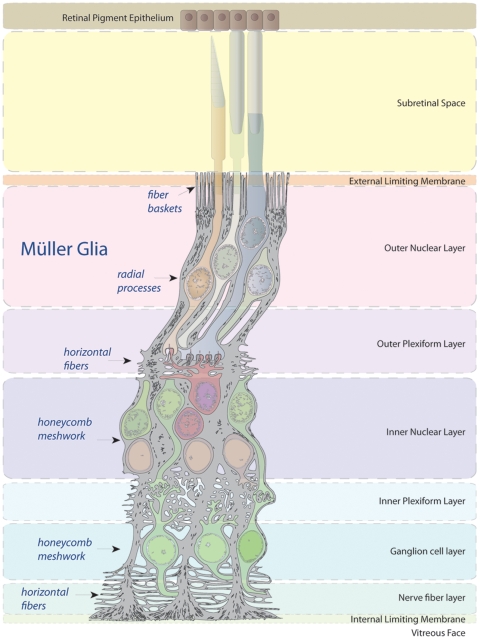
Müller glia in the retina. Illustration of Müller glia spanning the entire retina, where they ensheath all neuronal types from retinal ganglion cells (RGC) (bottom) to the photoreceptors. Modified from Histology of the Human Eye, an Atlas and Textbook. Hogan, Michael J., Jorge A. Alvarado, Joan Esperson Weddell. Philadelphia: W. B. Saunders, 1971.

Despite these benefits, intravitreal injections pose significant challenges. First, dilution of vector into the vitreal fluid leads to lower vector concentrations compared to subretinal injections, a particular concern for larger mammals. However, intravitreal vector administration into macaque eyes showed that AAV is still able to overcome this challenge and lead to retinal transduction [23]. An additional consideration is that the increase in vitreous viscosity in humans above the age of 40 could impair the diffusion of the virus in older patients' vitreous, though this concern is potentially offset by an increase in liquid vitreous localized directly in front of the retina [24] enabling one to inject into this fluid region and avoid the more viscous vitreous gel. In addition to these vitreal fluid barriers, treating photoreceptor degeneration in early stages of disease progression using gene delivered neurotrophic factors requires high efficiency vectors, but intravitreal injection of conventional AAV serotypes cannot mediate sufficient expression of these factors in proximity to the photoreceptors undergoing degeneration in the outer nuclear layer of the retina to achieve effective rescue of retinal function [25]. Infecting Müller glia, a cell that is accessible from the vitreous and transverses the entire thickness of the retina, would be ideal to mediate expression of secreted neurotrophic or anti-angiogenic factors throughout all layers of the retina (**Fig. 1**).

Müller cells serve numerous, significant physiological functions in the retina including but not limited to: metabolic support of neurons, K^+^ and water homeostasis, free radical scavenging and oxidative protection, neurotrophic secretion, and neurotransmitter and retinoid uptake and recycling [26]. They are thus in many ways analogous to astrocytes in the central nervous system (CNS), sharing many of the same cellular properties [27], [28]. Moreover, because Müller cells can survive under neurodegenerative conditions, they may mediate independent protection of the entire retina for a more extended period compared to a transduced damaged or dying neuron impacted by one of these diseases. As such, they represent an ideal candidate for viral gene therapy for secretion of neuroprotective factors.

Recently, the efficiency of this approach has been demonstrated as Müller cell secretion of neurotrophin-4 (NT-4) was shown to protect photoreceptors from oxidative stress in a mouse model of neovascularization through intravitreal injection [29]. However, the therapeutic effect of this approach can be enhanced using a more efficient AAV variant, as current AAV serotypes are limited in their efficiency and specificity for intravitreal Müller transduction [25], [29]. An AAV variant with greater Müller cell specificity would also allow the use of fewer viral particles to achieve the desired levels of expression and reduce the potential for ectopic transfection, important considerations to minimize the immune response to capsid proteins [15], [30].

Recently, we applied directed evolution to select variants from combinatorial libraries that demonstrate a diverse range of cellular tropisms *in vitro* and *in vivo* relative to their parent serotypes as well as variants with enhanced permissivity to astrocytes *in vivo* through selection in primary human astrocytes [14], [31]. Here, we further explore the utility of these astrocyte-permissive variants for intravitreal administration in the retina and identify a new AAV variant with enhanced and specific intravitreal Müller cell transduction *in vivo*. Efficient and specific intravitreal transduction of Müller cells by this vector will permit the development of more innocuous and effective treatments for retinal degenerative conditions through panretinal secretion of neuroprotective or anti-angiogenic factors, as well as new approaches to investigate fundamental questions in the biological role of this cell type in the retina.

## Results

### *In vivo* characterization of Müller cell permissive variants

We recently evolved several novel AAV capsids that efficiently transduced both primary human astrocytes *in vitro* and rat astrocytes *in vivo* using highly diverse AAV libraries (>10^7^) [14]. These variants were generated via multiple evolutionary rounds (i.e. diversification followed by positive selection for enhanced astrocyte transduction *in vitro*) with several distinct libraries: (1) an AAV2 random mutagenesis library generated via error prone PCR [32], (2) a random chimera AAV library generated by shuffling the *cap* genes of 7 natural human and non-human AAV serotypes [31], and (3) a novel AAV2 library with surface-exposed loops of the capsid library diversified based on a bioinformatics approach [14].

**Figure 2:**
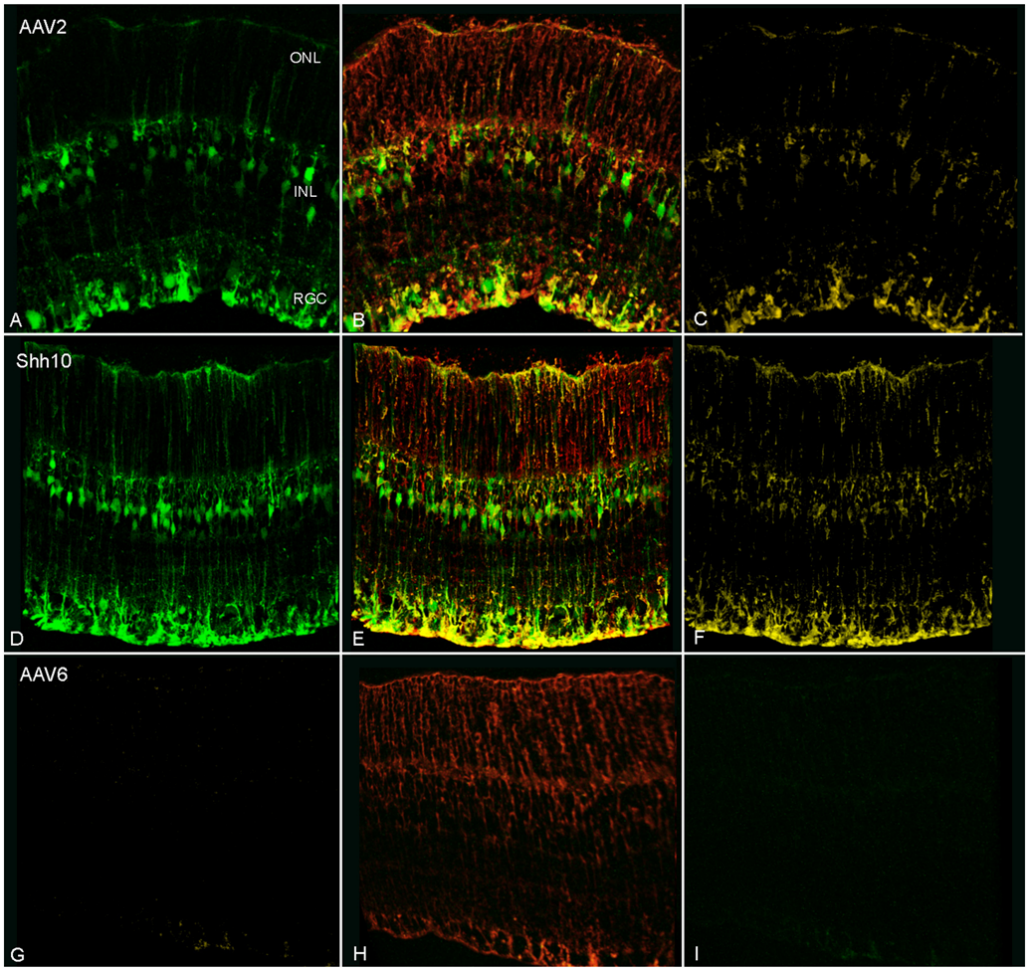
rShH10 expression following intravitreal injection in the adult rat retina. Confocal imaging of immunostained transverse retinal sections 3 weeks post-injection of 2.5×10^10^ viral particles (vector genomes) of dsCAG-GFP vectors with capsids from AAV2 (A–C), ShH10 (D–F), and AAV6 (G–I) (n = 6). Glutamine synthetase (GS) staining (red) (B,E,H) and visualization of colocalization (C,F,I) reveals more robust Müller cell expression by ShH10 (E, F) relative to AAV2 (B,C), whereas AAV6 shows no visible expression (G–I). Additionally, GFP expression shows specific transduction of Müller cells by ShH10 (D) compared to AAV2 (A), which exhibits considerably more transduction of retinal ganglion cells and interneurons.

**Figure 3:**
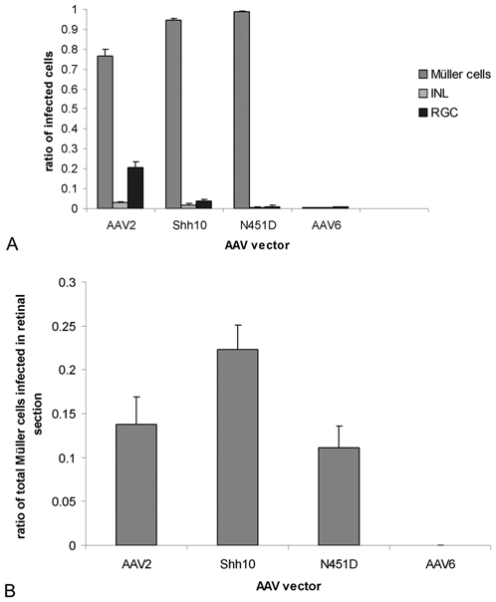
Transduction specificity and efficiency of ShH10. Representative retinal slices from injected eyes were quantified for the number of each cell type that was infected, as determined via GFP expression, to generate histograms comparing tropism profiles (A) and Müller transduction efficiencies (B) of rAAV2, rShH10, and rAAV6 dsCAG-GFP. Transduction efficiencies were calculated based on the ratio of Müller cells infected relative to the total number of Müller cells in a 10 µm transverse retinal slice (n = 6). Error bars represent standard deviation among sample population.

Due to the shared properties between astrocytes and Müller cells, we decided to explore the utility of these variants for intravitreal transduction of Müller cells. Here, the eight isolated mutants that demonstrated the greatest *in vitro* astrocyte infectivity (**Fig. S1**, [14]) were individually analyzed for the ability to transduce the retina from the vitreous using double-stranded (ds) AAV CAG-GFP vectors purified via iodixanol gradient ultracentrifugation and heparin affinity chromatography. Intravitreal injections of 2.5×10^10^ genomic particles revealed one previously unreported variant named ShH10, derived from an AAV6 parent serotype from the shuffled (ShH) library, that showed a dramatic increase in specificity and efficiency for Müller cells relative to controls at three weeks post-injection (**Figs. 2** **,** **3**). Interestingly, no other mutants demonstrated visible expression as determined by GFP fundus imaging and immunohistochemistry (data not shown). Recombinant ShH10 (rShH10) led to diffuse expression throughout the retina with a *highly* specific transduction profile of approximately 94% Müller cells, 2% interneurons, and 4% retinal ganglion cells (**Figs. 2** **,** **3** **,** **4**). In comparison, the parent vector, AAV6, showed very low transduction of the retina, and the related AAV2 vector showed a less specific retinal tropism with a transduction profile of approximately 76% Müller cells, 3% interneurons, and 21% retinal ganglion cells (**Figs. 2** **,** **3** **,** **4**). Quantification of transduction efficiencies revealed that ShH10 was approximately 62% more efficient at infecting Müller cells relative to AAV2, infecting 22% vs. 14% of total Müller cells respectively in transverse retinal slices (**Fig. 3b**).

**Figure 4:**
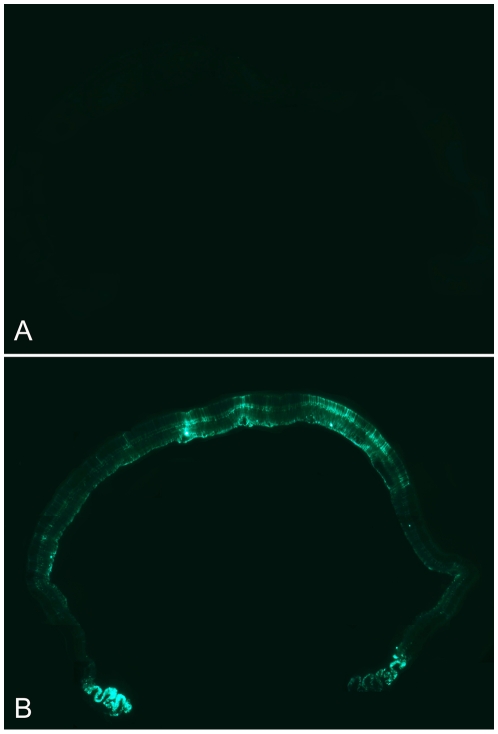
rShH10 expression in the whole retina following intravitreal injection. Fluorescence microscopy of transverse retinal sections from rShH10 dsCAG-GFP and rAAV6 dsCAG-GFP injected animals 3 weeks post-injection reveals broadly spread expression by ShH10 (B), with the most prominent expression localized at the injection site. AAV6 (A) shows no visible expression.

**Figure 5:**
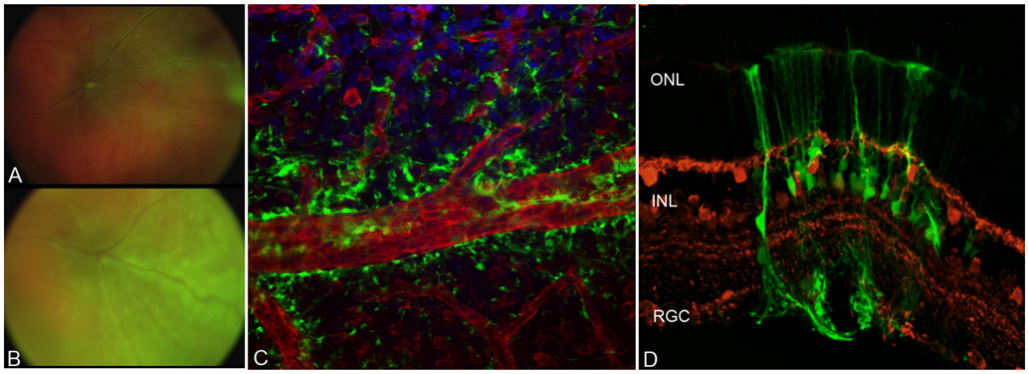
Retinal astrocyte infectivity of ShH10. Fundus imaging of rShH10 dsCAG-GFP injected animals at one week (A) reveals a characteristic expression pattern localized near major vasculature and the optic nerve, which subsequently shows spreading after three weeks (B). Closer examination by flatmount (C) through laminin (red) and DAPI (blue) staining reveals a strong localization of GFP expression along the edges of retinal blood vessels, areas dense in retinal astrocytes. Transverse sections (D) stained for calbindin (red), a marker of RGCs and interneurons in the retina, illustrate a local region of expression within retinal astrocytes and Müller cells ensheathing a blood vessel.

Temporal observation of ShH10 expression using fundus imaging, coupled with anti-laminin immunostaining of retinal flatmounts to visualize vasculature, also revealed a unique tropism for retinal astrocytes at earlier time points following injection (**Fig. 5**). Unlike Müller cells, retinal astrocytes are not derived from the retinal neuroepithelium, but serve some analogous roles in the retina including providing nutritional support to neurons, neurotransmitter metabolism, and ionic homeostasis [33]. They also serve as axonal glial sheaths for ganglion cells bodies and envelop the retinal vasculature, forming part of the blood-brain barrier. One week post-injection, fundus imaging revealed localized expression near those areas dense in retinal astrocytes, e.g. the optic nerve and along retinal vasculature (**Fig. 5a**). Additionally, transverse retinal sections showed that areas underlying major vasculature bore strong Müller expression (**Fig. 5d**). At later time points (2–3 weeks), expression became more evenly spread, but interestingly, those regions in proximity to vasculature ultimately maintained the strongest Müller cell expression (**Fig. 4**).

### Mutational analysis of ShH10

**Figure 6:**
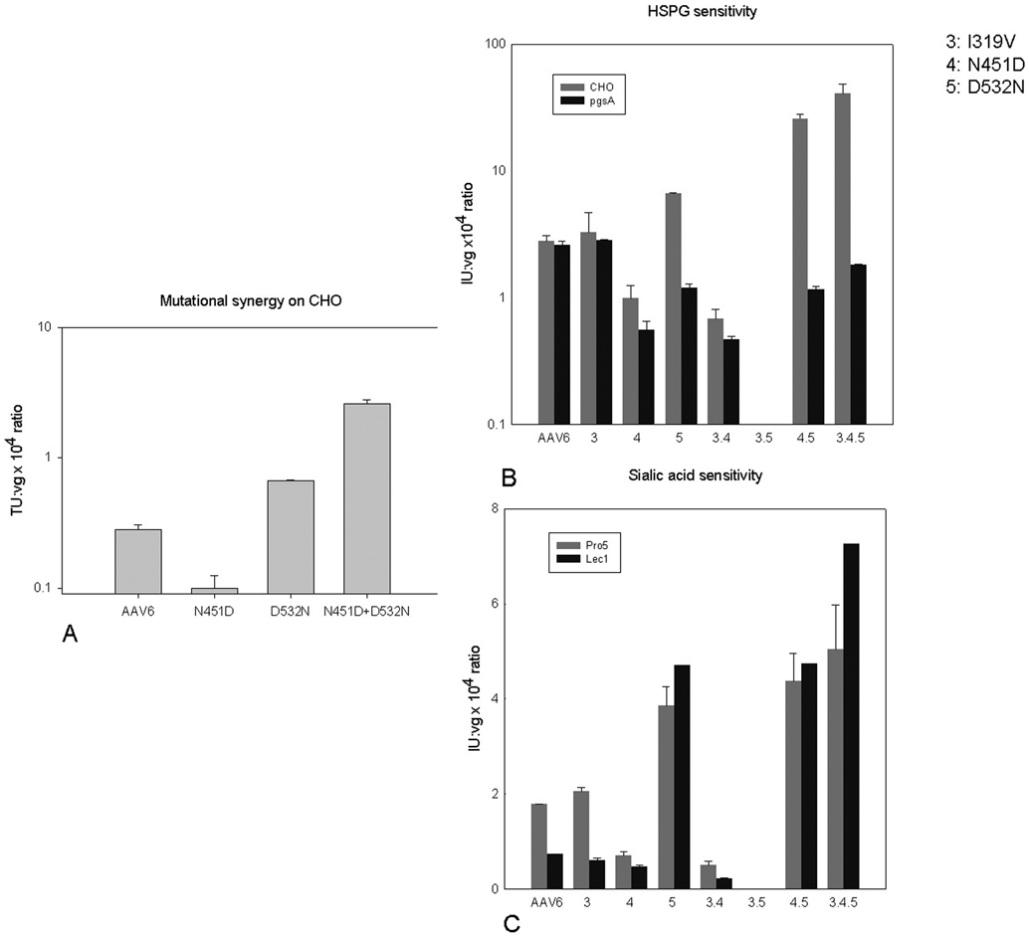
In vitro characterization of ShH10. (A) CHO cell transduction by rAAV6, rAAV6 N451D, rAAV6 D532N, and rAAV6 N451D+D532N carrying CMV-GFP. (B) CHO/PgsA transduction demonstrating the HSPG dependence of various permutations of the mutations that comprise ShH10. (C) Pro5/Lec1 transduction examining sialic acid dependence of various permutations of the mutations that compose ShH10.

**Figure 7:**
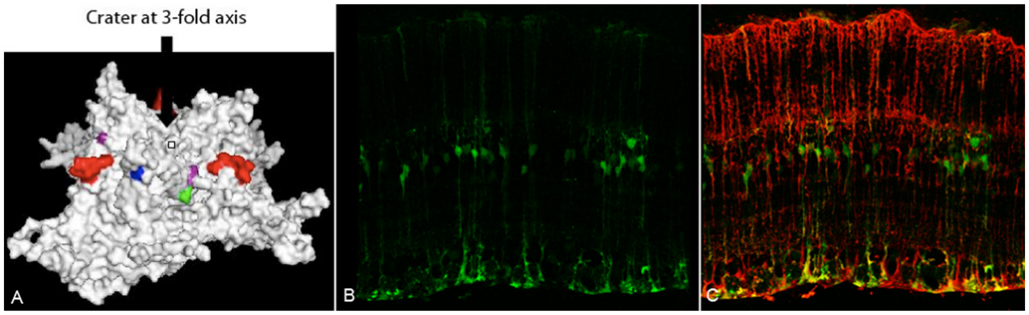
rAAV6 N451D expression following intravitreal injection. Confocal imaging of immunostained transverse retinal sections 3 weeks post-injection of rAAV6 N451D dsCAG-GFP (B, C). GFP expression analysis (B) and overlay with GS (C) reveal this mutant to be sufficient for an intravitreal Müller infection, though at a reduced efficiency relative to ShH10 (fig. 1D, F). Mapping of this mutation onto the AAV6 capsid subunit VP3 (A) (blue) shows its location near the three-fold axis of symmetry of the assembled capsid. Three-dimensional models of the AAV6 VP3 subunit were generated using Swiss Model with the coordinates of AAV2 (Protein Databank accession no. 1LP3) supplied as a template and images were rendered in Pymol and Rasmol. Additionally, the D532N mutation (green) maps near the HSPG-binding domain (purple) [35].

ShH10 is highly Müller cell selective, while AAV6 yields no detectable retinal expression upon intravitreal administration, yet intriguingly ShH10 differs from AAV6 at only four residues: I319V, N451D, D532N, and H642N (**Fig. S1**). To analyze the contributions of each of these mutations to ShH10's novel phenotype, single point mutants and all potential double mutants were generated from the AAV6 *cap* gene via site-directed mutagenesis. Each resulting variant was used to package rAAV-CMV-GFP and was purified via iodixanol gradient ultracentrifugation. To characterize the *in vitro* infectivity of these mutants, and in particular their glycan dependence in light of the substantial role proteoglycans and glycoproteins play in AAV transduction [4], [5], [13], we analyzed their relative transduction efficiencies on a panel of cell types: Pro5, a Pro5 mutant (Lec1) deficient in N-linked sialic acid, CHO, and a CHO derivative (pgsA) deficient in all glycosaminoglycans [34]. AAV6 exhibited a dependence on N-linked sialic acids for efficient transduction, as previous studies have indicated (**Fig. 6c**) [13] . However, the N451D mutation decreased the viral dependence on N-linked sialic acids, and the D532N mutation increased the viral transduction in the absence of N-linked sialic acids (**Fig. 6c**). This D532N mutation, located near the HSPG binding domain of the AAV6 capsid [35], may enable the virus to utilize a transduction pathway distinct from AAV6 (**Fig. 7**).

Whereas AAV6 does not utilize HSPG for transduction (**Fig. 6b**) [13] , several of the ShH10 mutations confer a new dependence on HSPG. Intriguingly, AAV6 N451D exhibited lower transduction levels relative to AAV6 in CHO cells, but when coupled with the D532N mutation was more infective than either AAV6 or AAV6 D532N (**Fig. 6a**). Comparing infection efficiencies among the single point mutants between CHO and pgsA cells, cell lines containing and lacking HSPG respectively, AAV6 D532N was the only mutant to exhibit a substantial HSPG dependence, which became more pronounced when coupled with mutations I319V and N451D (**Fig. 6b**). The enhanced infectivity of ShH10 is thus likely due to a synergy between mutations that in part augments HSPG affinity as suggested by the heparin affinity chromatogram (Fig. S2). To determine whether AAV6 mutations that enhance infectivity also function *in vivo*, equal titer intravitreal injections of 5×10^9^ genomic particles of recombinant vector mutants carrying dsCAG-GFP revealed that that only AAV6 N451D was sufficient to confer the intravitreal Müller tropism. This mutant was considerably more efficient than AAV6 on Müller cells, though only half as efficient as ShH10 (**Fig. 3** **,** **7b**).

## Discussion

Our results demonstrate that the selection on primary human astrocytes from our previous study [14] was sufficient to produce a variant that overcomes intraocular barriers to infection, apparently due to the cellular similarities between Müller glia and astrocytes. These similarities may be based on their shared cellular lineages, proteomic profiles, cellular polarity, and functionally analogous roles in the CNS [26], [27], [36], [37]. Interestingly, primary astrocytes may have even been more suitable as a selection model *in vitro* in lieu of directly selecting against primary Müller cells, as proteomic profiling studies of primary Müller cells in culture revealed a loss of cellular identity with significant shifts in expression that suggested a dedifferentiation into a fibroblast-like phenotype [38]. Characterizations of astrocytes in culture have pointed to fewer changes, thus making this cell type relatively closer to its initial *in vivo* state [39].

The *in vitro* evolution from our previous study selected for variants with the capacity to bind to the glial cell surface and traffic to the nucleus [14]. This may, in part, explain why the majority of variants were unable to overcome the additional extracellular barriers present in the retina *in vivo* [40]. However, one of the variants, ShH10, showed strong expression following intravitreal injection. Interestingly, sequence analysis (**Fig. S1**) reveals that ShH10 does not share mutations with the other evolved variants except for mutation H642N with variant ShH13, which has been shown in our previous work to have an enhanced tropism for Müller cells upon subretinal injection [14]. However, interestingly, ShH13 shows no expression with intravitreal administration (data not shown).

Importantly, in beginning to mechanistically investigate viral infection in the retina, our previous studies have shown that AAV binding at the inner limiting membrane (ILM) is implicated in mediating transduction via intravitreal injection of AAV serotype 2, and to a lesser extent 8 and 9 [40]. The ILM is a meshwork of extracellular matrix proteoglycans located at the interface of the vitreous and the retinal ganglion cell (RGC) layer and Müller cell endfeet (**Fig. 1**), and it constitutes one of the first physical barriers encountered by intravitreally injected virus. Receptors such as HSPG and the 32 k laminin receptor that are abundant at this site may allow binding of AAV serotypes 2, 8, and 9, whereas AAV serotypes such as 1 and 5 that bind sialic acid, which is not found at the ILM, show no expression in the rat retina following intravitreal administration [15], [25], [40]. Once binding to the ILM occurs, AAV then needs to overcome additional diffusional and trafficking barriers in the retina to achieve efficient transduction. In this respect, although AAV6 can infect cells upon subretinal injection [14], its subretinal tropism is largely limited to retinal pigment epithelia (RPE) and photoreceptors, and it completely lacks the capacity to infect retinal cells upon intravitreal injection (**Fig. 2**). These results suggest two distinct obstacles in AAV6's inability to infect Müller cells intravitreally: a marginal capacity to first bind to the ILM via proteoglycan interactions and a limited ability to then overcome Müller cell-surface and/or intracellular barriers.

In first binding to the ILM, the presence of high levels of heparan sulfate in the proteoglycan matrix of the ILM may mediate viral accumulation at this site and act as a ‘sink’ [40]. Although this may sequester AAV particles, it may also effectively localize the virus and prevent it from being cleared from the vitreous via the trabecular meshwork, thereby facilitating subsequent transduction [41]. Although wild-type AAV6 does not depend on HSPG for infection [13], ShH10 exhibits a newly conferred HSPG dependence, allowing it to better bind and traverse the ILM (**Fig. 6b**). Preliminary results indicate that the D532N mutation (shown in green in **Fig. 7a**) may be responsible for this dependence, as the D532N mutant exhibits enhanced viral binding to heparin and significantly increased transduction of CHO cells (data not shown and **Fig. 6a**, **S2**). This mutation lies next to the K531 residue (shown in purple in **Fig. 7a**) that has been shown to confer heparin binding to wild-type AAV6 [35]. The loss of negative charge in the D532N mutant may facilitate more efficient electrostatic interactions between K531 and the negatively charged sulfate groups present within HSPG. AAV2's higher HSPG affinity, but lower transduction efficiency, relative to ShH10's (**Fig. S2**) may then suggest a potential dueling role in achieving optimum transduction, as too high of an affinity may also encumber efficient viral penetration in the retina.

Once it traverses the ILM, ShH10 must bind Müller cells. Recent studies have implicated the epidermal growth factor receptor (EGFR), which is expressed on the surface of Müller cells, as a coreceptor for AAV6 transduction [42], [43]. However, since AAV6 is largely refractory to intravitreal transduction and has a marginal ability to transduce Müller cells subretinally [14], ShH10's novel tropism may then partly result from an improved binding affinity for EGFR (or another co-receptor) to enable Müller cell binding and endocytosis.

Preliminary results suggest that the N451D mutation may partly be responsible for such potentially improved receptor interactions, as this substitution is sufficient to confer a limited Müller cell intravitreal infectivity (**Fig. 7**). The asparagine to aspartic acid mutation places a negative charge near the tip of the 3-fold axis of symmetry on the AAV capsid (**Fig. 7a**), an area implicated in receptor binding on the AAV2 capsid [5]. Intriguingly, the N451D mutation (shown in blue in **Fig. 7a**) alone decreased viral transduction of CHO and Pro5 cells (**Fig. 6**), but when this mutation is combined with the D532N change, viral transduction of CHO cells is synergistically improved. This synergism may then reflect an improved receptor/co-receptor relationship, with the D532N mutation negating the detrimental aspects of the N451D mutation while concomitantly enhancing binding to a primary proteoglycan receptor (i.e. HSPG).

ShH10 exhibited enhanced transduction of Müller cells underlying areas of retinal vasculature, suggesting that its infectivity of retinal astrocytes may also offer a possible mechanistic explanation for its tropism. Whether this is just a result of commonalities between these glial subtypes that allow for ShH10 permissivity in both or whether infection of one type may facilitate adjacent infection of the other through localized binding remains to be analyzed. Regardless, this property would be useful in infecting the activated Müller cells in neovascularized tissue to deliver neurotrophic factors to sites of vascular abnormalities [29].

Overall, efficient and selective intravitreal transduction of Müller cells by AAV may provide safer and more effective treatments for retinal degenerative conditions through secretion of neuroprotective factors, while also enabling basic investigations to gain a greater understanding of Müller cell physiology. For instance, the uncertain role of Müller cells in the visual cycle of cone photoreceptors can be examined *in vivo* through Müller-specific AAV-mediated RNAi-knockdown of factors implicated in 11-cis-retinol generation [44]. Likewise, in a similar approach, the undefined role of these cells in regulating the fluid dynamics of the retina could be further explored by specifically modulating Müller aquaporin (AQP) levels [45].

For future studies, the use of highly diverse AAV libraries coupled with *in vivo* selections could provide a more powerful approach to directly target specific cell types in the retina. For example, the targeting of engineered light gated channels to specific subpopulations of retinal cells, such as ON bipolar cells or ON/OFF retinal ganglion cells [46], may restore functional vision in individuals with a severely degenerated ONL by reestablishing downstream retinal circuitry. Likewise, targeted delivery to photoreceptors may enable additional strategies for neuroprotective therapy.

## Methods

### Generation of rAAV vectors

Vectors were produced by the plasmid co-transfection method [14], and the resulting lysates were purified via iodixanol gradient ultracentrifugation as previously described [31]. This fraction was then passed through a heparin column, which was washed with 5 mL PBS and eluted with 5 mL of a 1 M NaCl solution. The resulting viral fractions were desalted and concentrated with Amicon Ultra-15 Centrifugal Filter Units to a final volume of 200 µl. Vector was then titered for DNase-resistant vector genomes by real time PCR relative to a standard.

### Intraocular administration routes

Adult wild type Spaque Dawley rats were used for all studies. All animal procedures were conducted according to the ARVO Statement for the Use of Animals and the guidelines of the Office of Laboratory Animal Care at the University of California, Berkeley. Before vector administration, rats were anesthetized with ketamine (72 mg/kg) and xylazine (64 mg/kg) by intraperitoneal injection. An ultrafine 30 1/2-gauge disposable needle was passed through the sclera, at the equator and next to the limbus, into the vitreous cavity. Injection of 5 µl, containing 1−5×10^12^ vg/ml of AAV dsCAG-GFP, was made with direct observation of the needle in the center of the vitreous cavity.

### Fundus photography

Fundus imaging was performed one to eight weeks after injection with a fundus camera (Retcam II; Clarity Medical Systems Inc., Pleasanton, CA) equipped with a wide angle 130° retinopathy of prematurity (ROP) lens to monitor eGFP expression in live, anesthetized rats. Pupils were dilated for fundus imaging with phenylephrine (2.5%) and atropine sulfate (1%).

### Cryosections

One to eight weeks after vector injection, rats were humanely euthanized, the eyes were enucleated, a hole was introduced in the cornea, and tissue was fixed with 10% neutral buffered formalin for 2–3 hours. The cornea and lens were removed. The eyecups were washed in PBS followed by 30% sucrose in the same buffer overnight. Eyes were then embedded in optimal cutting temperature embedding compound (OCT; Miles Diagnostics, Elkhart, IN) and oriented for 10 µm thick transverse retinal sections.

### Immunolabeling and histological analysis

Tissue sections were rehydrated in PBS for 5 min, followed by incubation in a blocking solution of 1% BSA, 0.5% Triton X-100, and 2% normal donkey serum in PBS for 2–3 hours. Slides were then incubated with commercial monoclonal antibodies raised against glutamine synethetase in rabbit (Sigma G2781) at a 1∶3000 dilution, calbindin (Abcam ab11426-50) in rabbit at a 1∶1000 dilution, vimentin in mouse (Dako M0725) at a 1∶1000 dilution, or laminin in rabbit (Sigma, L9393) at 1∶100 in blocking solution, overnight at 4°C.

The sections were then incubated with Cy3-conjugated secondary anti-rabbit or anti-mouse antibody (Molecular Probes) at a 1∶1000 dilution in blocking solution for 2 hours at room temperature. The results were examined by fluorescence microscopy using an Axiophot microscope (Zeiss, Thornwood, NY) equipped with a Xcite PC200 light source and QCapturePro camera or a confocal microscope (LSM5; Carl Zeiss Microimaging). Transduction profiles were analyzed by counting individual cells from whole retinas in 15 µm cryosections (n = 6) using fluorescence microscopy. Efficiencies were calculated by dividing the total number of these transduced Müller cells by the total number of Müller cells in the retinal slice (mm) in which these cells were present (n = 6).

### *In Vitro* Transduction Analysis

Transduction studies using rAAV CMV-GFP were performed with 5×10^4^ cells (CHO, pgsA, Pro5, and Lec1) in 12-well plates. Cells were transduced with rAAV GFP vectors at a gMOI of 10^3^–10^5^ (n = 3), and the percentage of GFP-expressing cells was determined by flow cytometry 48 hours post-infection.

## Supporting Information

Figure S1 Sequence analysis of novel AAV variants. Sequence comparison between ShH10 and ShH13 along with parent AAV serotypes 2 and 6.
(0.03 MB PDF)

Figure S2 Heparin binding affinity of ShH10, AAV2, and AAV6. Elution profile from a heparin column for ShH10, AAV2, and AAV6. Y-axis values represent the fraction of virus eluted, and the X-axis represents the concentration of NaCl in the eluant (mM).
(8.32 MB TIF)
