# Supplementary figures and images for: A Novel Adeno-Associated Viral Variant for Efficient and Selective Intravitreal Transduction of Rat Müller Cells
Source: PLoS One. 2009 Oct 14;4(10):e7467. doi: 10.1371/journal.pone.0007467 (PMC2758586; doi:10.1371/journal.pone.0007467)

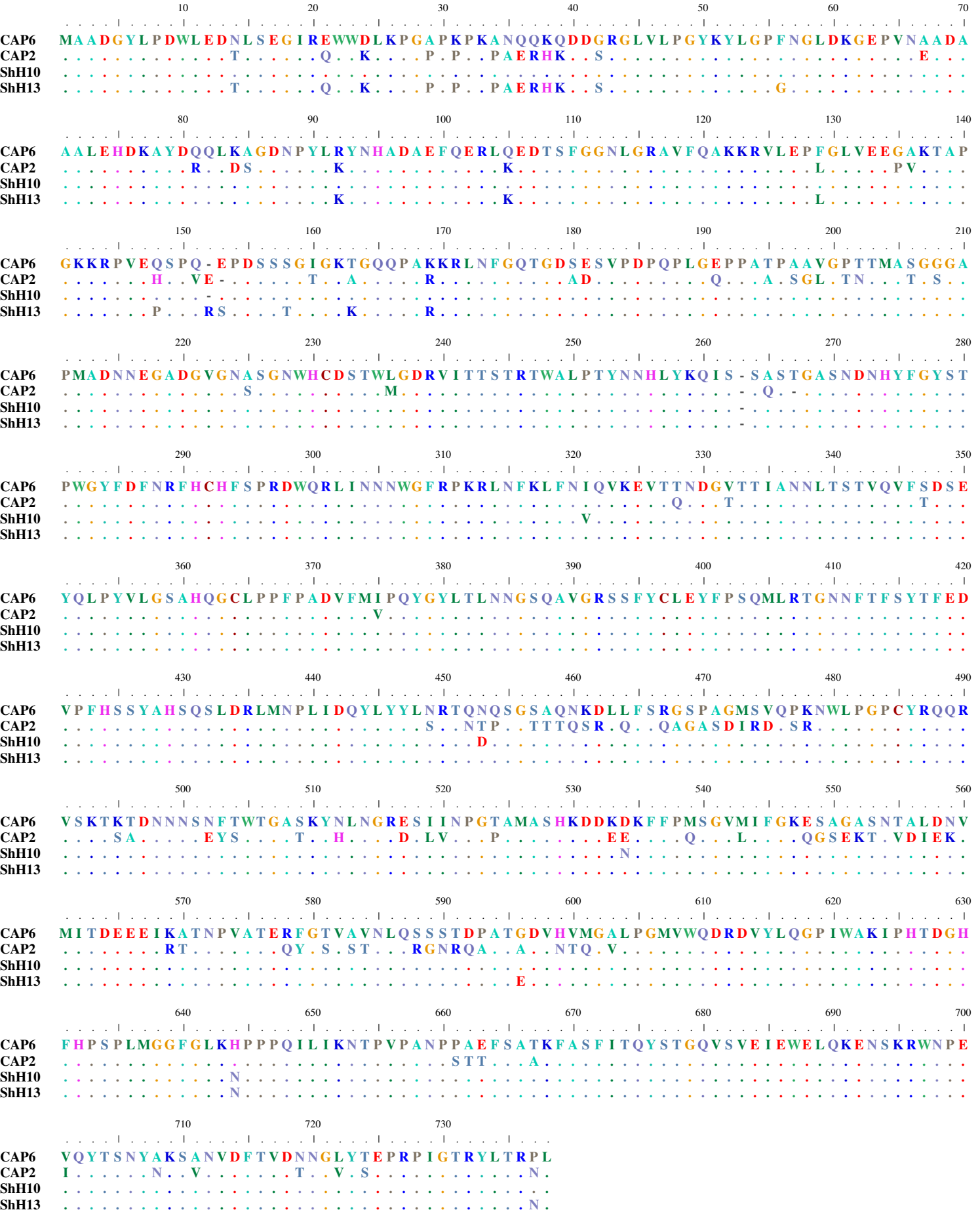

Supplement: Figure S1 — Sequence analysis of novel AAV variants. Sequence comparison between ShH10 and ShH13 along with parent AAV serotypes 2 and 6. (0.03 MB PDF) [file pone.0007467.s001.pdf]

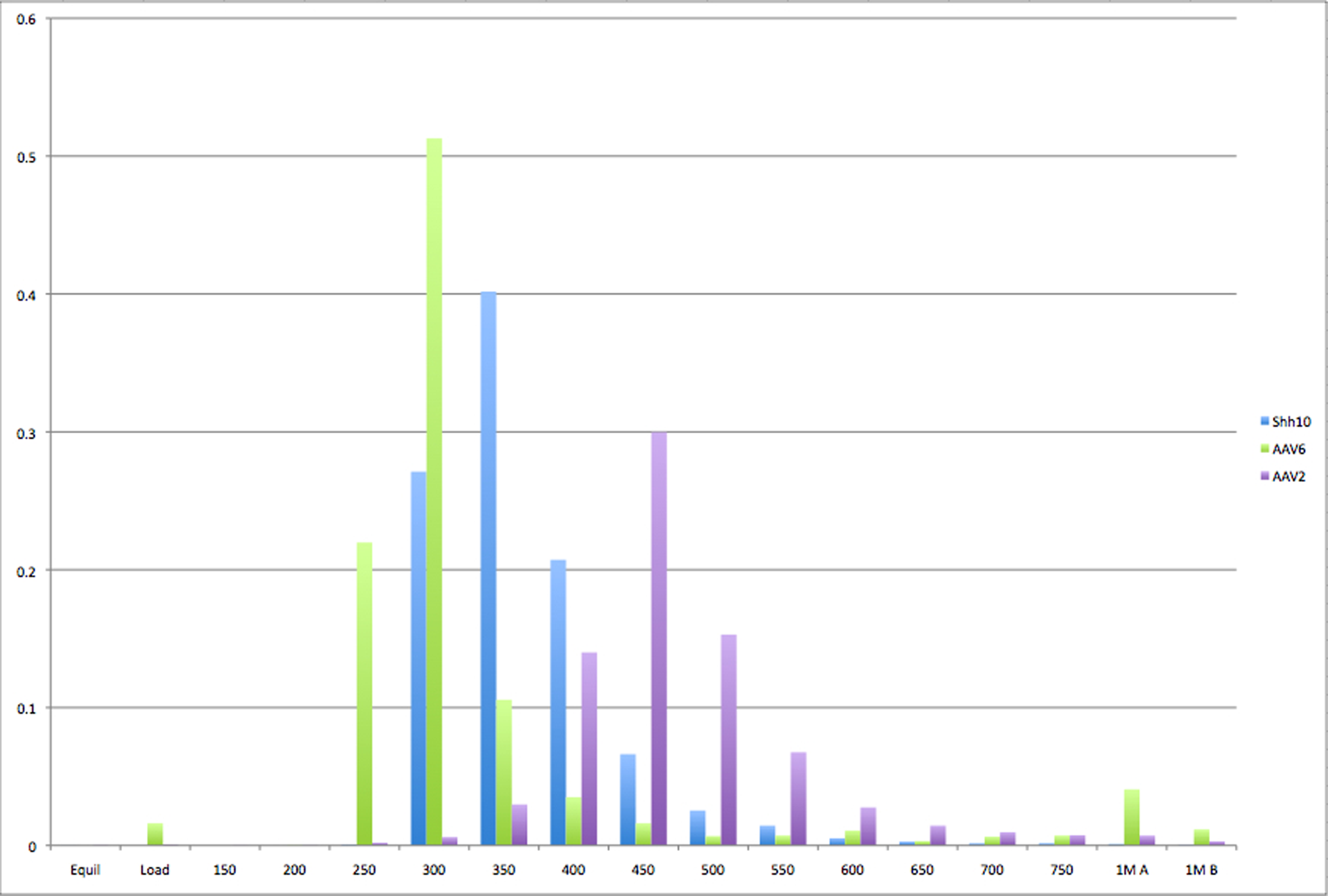

Supplement: Figure S2 — Heparin binding affinity of ShH10, AAV2, and AAV6. Elution profile from a heparin column for ShH10, AAV2, and AAV6. Y-axis values represent the fraction of virus eluted, and the X-axis represents the concentration of NaCl in the eluant (mM). (8.32 MB TIF) [file pone.0007467.s002.tif]
